# Cœur à six cavités chez un enfant de 11 ans

**DOI:** 10.11604/pamj.2014.17.156.2267

**Published:** 2014-03-04

**Authors:** Euloge Yiagnigni Mfopou

**Affiliations:** 1 Hôpital Central de Yaoundé, Service de Cardiologie, Yaoundé, Cameroun

**Keywords:** Cœur, cavités cardiaques, malformation, heart, cardiac cavities, malformation

## Image en médecine

Nous vous présentons cet article montrant le coeur d'un enfant de 11 ans bien portant sur plan physique et psychique, présentant une duplication totale bien segmentée du ventricule droit et de l'oreillette droite par un anneau issu de la duplication de l'anneau tricuspidien. Cette malformation a été découverte dans le cadre d'investigation des douleurs thoraciques atypiques. Nous n'avons pas retrouvé dans la littérature la description d'un cas pareil. C'est une variante clinique compatible avec la vie et sans impact sur le développement physico-psychique de la personne porteuse. L'exploration échographique n'a pas permis de bien décrire la circulation sanguine dans cette partie du coeur. Cet article décrit un autre type de malformation compatible avec la vie. La description complète n'a pas été faite car nous sommes limités par les moyens d'exploration.

**Figure 1:**
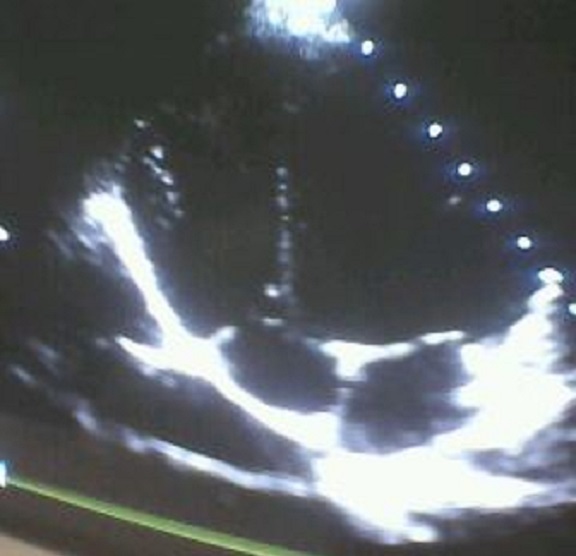
Cœur à six cavités chez un enfant de 11 ans (duplication du ventricule et oreillette droit)

